# Experimental investigation of factors affecting microbial liquefaction mitigation in coastal liquefied sand

**DOI:** 10.3389/fmicb.2026.1800127

**Published:** 2026-03-20

**Authors:** Jun Hu, Mengyi Liu, Jixun Ren, Yu Li, Houhai Yuan

**Affiliations:** 1 State Key Laboratory of Tropic Ocean Engineering Materials and Materials Evaluation, Hainan University, Haikou, China; 2 School of Civil Engineering and Architecture, Hainan University, Haikou, China; 3 Marine Science and Technology Collaborative Innovation Center, Hainan University, Haikou, China; 4 State Key Laboratory for Tunnel Engineering, School of Civil Engineering, Shandong University, Jinan, China; 5 Hainan Nonferrous Engineering Survey and Design Institute Co., Ltd., Haikou, China

**Keywords:** coastal liquefied sand, environmentally friendly technique, gas-production efficiency, microbial, MIDP

## Abstract

**Introduction:**

Microbially induced desaturation and precipitation (MIDP) is a promising environmentally friendly technique for improving liquefiable coastal sands; however, the key factors governing microbial gas-production efficiency and their governing patterns remain insufficiently understood.

**Methods:**

To clarify the gas-production characteristics of *Pseudomonas stutzeri* for potential MIDP application in coastal liquefied sand treatment, this study systematically investigated the effects of temperature, pH, bacterial concentration (OD_600_), and nitrate concentration (KNO_3_) using a four-factor, five-level orthogonal experimental design under controlled laboratory conditions.

**Results:**

The results show that gas-production efficiency increases markedly with temperature from 10 to 30 °C. Increasing pH from 5 to 9 enhanced the gas-production rate by 2.3-fold. As OD_600_ increased from 0.05 to 0.25, gas production first improved and then declined, with the optimum performance observed at OD_600_ = 0.10. Among the investigated factors, KNO_3_ concentration was identified as the most influential variable controlling gas production by *Pseudomonas stutzeri*.

**Discussion:**

These findings provide an experimental basis for optimizing the application of MIDP in the anti-liquefaction treatment of coastal liquefiable sand.

## 1 Introduction

In recent years, with the rapid development of social economy and population explosion in coastal areas of China, the scope of urban construction continues to expand. In the process of engineering construction, various kinds of adverse geological phenomena are often encountered, in which the liquefaction stratum of sand and soil, as a typical geotechnical engineering problem, is particularly prominent in the coastal area. Under the action of earthquakes, the liquefaction of foundation sandy soil shows the flow characteristics leading to the loss of bearing capacity, which in turn triggers the tilting of the upper building structure, collapse and uneven settlement and other damages. Therefore, it is of great significance to take effective measures to deal with liquefied sandy soil in order to ensure the safety and stability of buildings in coastal areas and to prevent disasters caused by liquefied foundations. In engineering practice, traditional liquefied sandy soil treatment methods such as vibroplaning method, gravel compaction pile method, dynamic consolidation method and sheet pile enclosure method have become the most widely used technical means in the treatment of liquefied sandy soil foundations due to the advantages of simple operation, high construction efficiency (Wang et al., 2022; Xue et al., 2024; Jia et al., 2023; Qiu et al., 2024; Zhu et al., 2021). However, its limitations should not be ignored, such as high energy consumption and significant environmental disturbance effects are particularly prominent. In particular, the traditional process often requires the injection of highly polluting chemical slurry, which is prone to cause secondary pollution to the surrounding soil and groundwater environment. Based on this situation, scholars at home and abroad are committed to research and development of eco-friendly liquefied sand foundation treatment technology.

In recent years, scholars at home and abroad have introduced microbial technology into the field of foundation treatment of liquefied sandy soils, and the anti-liquefaction performance of sandy soils has been significantly improved by biological improvement (Ferris et al., 1997; He, 2013). Among them, microbially induced calcium carbonate precipitation (MICP) technology is most widely used. The technology is based on the biomineralization of urease bacteria, which generates carbonate ions (CO_3_^2−^) by catalyzing the hydrolysis of urea, which combines with calcium ions (Ca^2+^) to form a cemented calcium carbonate (CaCO_3_) precipitate. At present, this technology has formed systematic research results in geotechnical engineering fields such as soil reinforcement, fissure sealing and slope stabilization (Cui et al., 2017; Yuan et al., 2024; Arpajirakul et al., 2021), showing good engineering application prospects. Another innovative approach is the microbially induced desaturation and precipitation (MIDP) technology, which generates nitrogen gas through a denitrifying bacteria-driven biological denitrification process that utilizes a gaseous phase change to reduce sand saturation. The chemical equation is:


$${\mathrm{5C}}_{5}H_{7}{C_{2}}^{-}+18{{\mathrm{NO}}_{3}}^{-}\to 25{\mathrm{CO}}_{2}↑+9\;N_{2}↑+14H_{2}O+18{\mathrm{OH}}^{-}$$

This technique was firstly proposed by Rebata-Landa and Santamarina (2012), the micro- and nano-sized nitrogen bubbles generated by MIDP have low solubility characteristics, which can be long termly endowed in the pore network of sandy soil. Under seismic loading, the bubbles consume energy through compression and deformation, effectively suppressing the accumulation of excess pore water pressure, thus realizing the improvement of the anti-liquefaction performance of sandy soil (O’Donnell et al., 2017). This technique provides a new bio-reinforcement pathway for the treatment of liquefied sandy soils. He (2013) investigated the size of the bubbles by visual inspection and CT scanning. He and Chu (2014) found that microbial bubbles had an average diameter of about 0.1–0.3 mm in the pores of the sand samples and were uniformly distributed. He and Chu (2014) found that the static liquefaction resistance could be doubled and saturation decreased to 88–95% by microbial desaturation by means of single-triaxial tests. In addition, He et al. (2013) have conducted shaking table tests to evaluate the liquefaction resistance of liquefied sandy soils after microbial gas desaturation. He (2013) demonstrated that microbial gas desaturation with a saturation degree of 95% was effective in avoiding liquefaction of loose sandy soils in saturated condition. Wu (2015) found that the excess water pressure decreased significantly when the saturation of sand samples was reduced to 90%. Peng et al. (2021) found that the maximum shear strain decreased with the decrease of saturation for the same relative density and seismic vibration acceleration. Wang (2020) conducted shaking table tests on inclined foundations and found that the cumulative lateral strain decreases with decreasing saturation. He attributed this to the presence of effective stresses in microbial desaturation that resist the sliding tendency during vibration. Baziar et al. (2022) also found that liquefaction-induced lateral dilatation could be reduced to 1/3 to 1/2 of its original size after microbial desaturation.

In this study, *Pseudomonas stutzeri*, which has efficient denitrification characteristics, was selected to prepare a standard bacterial solution, and the performance of microbial gas production in liquefied sandy soil was investigated by a 4-factor, 5-level orthogonal test (temperature, pH, OD_600_ and KNO_3_ concentration).

## 2 Materials and methods

### 2.1 Test materials

#### 2.1.1 Experimental bacteria

*Pseudomonas stutzeri* was purchased from Beijing Microbiological Culture Collection Center (BJMCC) with the original number of JCM 5965. P*seudomonas stutzeri* is one of the most widely studied microorganisms. It is very common in soil, has strong denitrification ability, and is a nitrogen-fixing bacterium that can convert nitrogen ions in the environment into nitrogen gas through denitrification reaction.

#### 2.1.2 Culture media

The culture medium used was the LB medium recommended by the manufacturer. Luria-Bertani (hereinafter referred to as LB) medium was used for microbial activation and expansion culture, which consisted of peptone 5.0 g, beef extract 3.0 g, and NaCl 5.0 g. It was added with ionized water to 1 L, and then sterilized by adjusting the pH to about 7.0. The LB medium was transferred to a conical flask and sterilized at 115 °C for 30 min in an autoclave. After sterilization was completed, 5 mL of bacterial solution was inoculated into 250 mL of LB medium cooled to room temperature, and the test bacterial solution was obtained by expanding the culture for 24 h at 30 °C, 190 rpm in a constant temperature oscillator.

Each liter of denitrification medium consisted of 0.2 g magnesium sulfate.

(MgSO₄·7H₂O), 1 g dipotassium hydrogen phosphate (K₂HPO₄), 5 g sodium citrate (C₆H₅Na₃O₇) as the carbon source, and potassium nitrate (KNO₃) as the nitrogen source at 1.0, 1.5, 2.0, 2.5, or 3.0 g, respectively.

#### 2.1.3 Experimental sand

**Figure 1:**
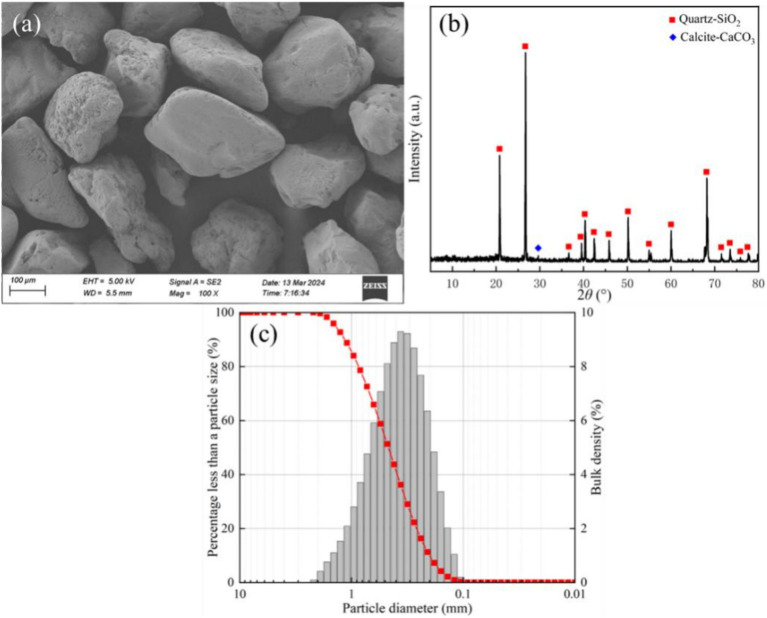
**(a)** SEM image of sea sand; **(b)** XRD image of sea sand; **(c)** Particle size distribution curve of sea sand.

The sea sand for the test was taken from Jiangdong New District, Haikou City, and the micro-morphology, material composition and particle size distribution are illustrated in Figure 1. From Figures 1a,b, it can be seen that the surface of the sea sand is smooth and the main component is SiO_2_. This sea sand has an effective grain diameter d_10_ = 0.26, a median grain diameter d_30_ = 0.32, a limiting grain diameter d_60_ = 0.40, coefficient of inhomogeneity C_u_ = 1.54, coefficient of curvature C_c_ = 0.98, a specific gravity of soil particles of 2.65, and a poorly graded sand.

### 2.2 Test method

#### 2.2.1 Microbial growth assessment

**Figure 2:**
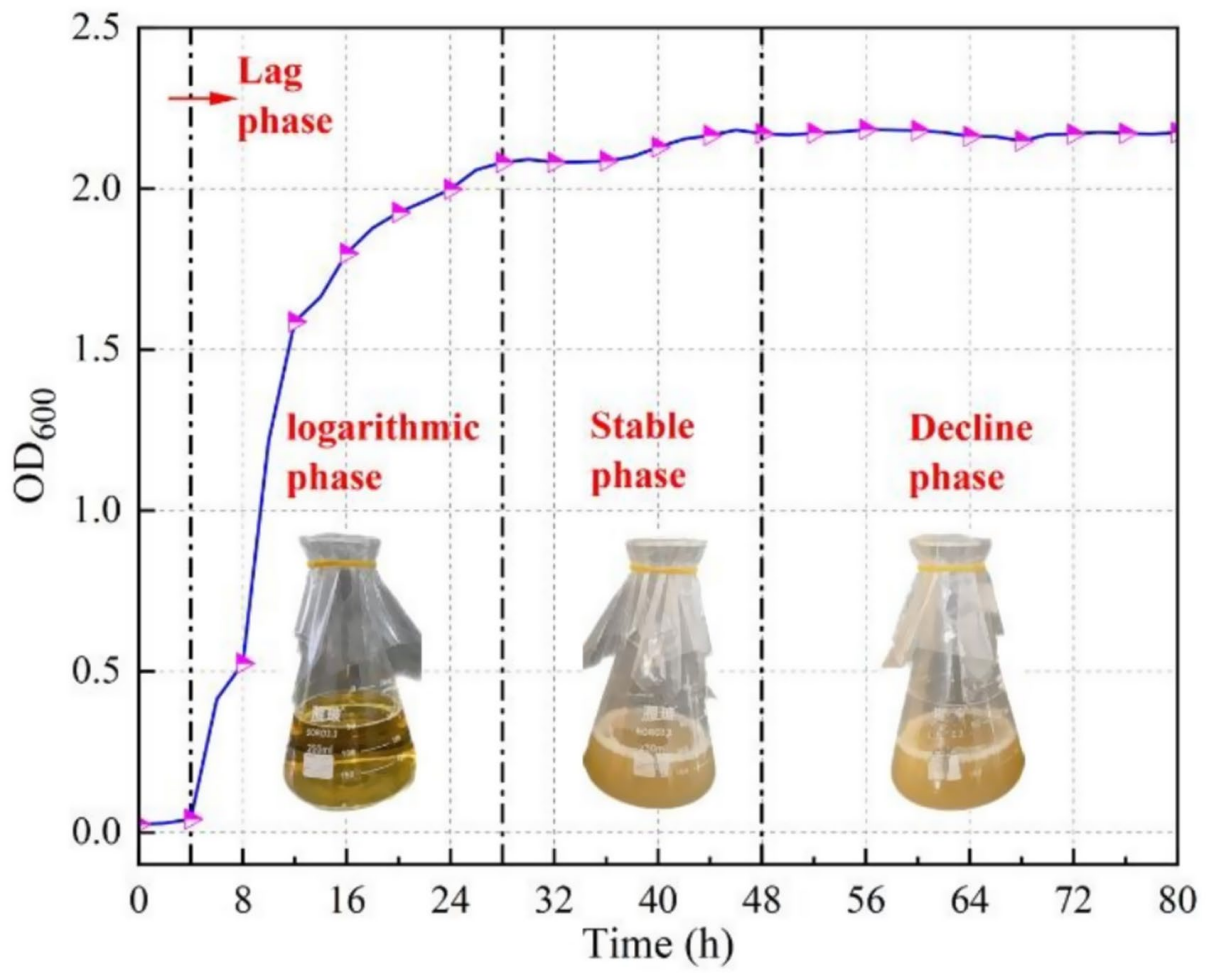
Microbial growth curve.

In order to understand the relationship between the growth time and activity of the microorganisms, *Pseudomonas stutzeri* was incubated for 80 h. Two milliliter of bacterial solution was taken in 2 cuvettes at 2 h intervals to measure its OD_600_ and recorded. The microbial growth curve is illustrated in Figure 2. From the figure, it was observed that a typical microbial growth cycle is divided into four phases, lag phase, logarithmic phase, stable phase and decline phase. 0–4 h is the lag phase. During this period, the bacterium increases in size and metabolism is active. Sufficient enzymes, coenzymes and intermediate metabolites are synthesized and accumulated for the division and reproduction of bacteria. 4 ~ 28 h is logarithmic phase. In this period, the bacterial growth rate is the fastest, the time required for each division is the shortest, the enzyme system is active, and the metabolism is vigorous, which is a good material for metabolic and physiological research. After 28 h, it was the stable phase. The growth rate constant was about equal to 0, the number of new cells and the number of dead cells were almost equal, and the two were in dynamic equilibrium. The number of viable bacteria remained relatively stable and reached the highest level, and the bacterial production also reached the highest point. The rate of bacterial division decreased, the generation time was gradually prolonged, the cell metabolic vigor gradually decreased, and changes in morphology and physiological characteristics began to appear. After about 50 h, the microorganisms entered into decline phase.

#### 2.2.2 Gas-producing performance test

**Figure 3:**
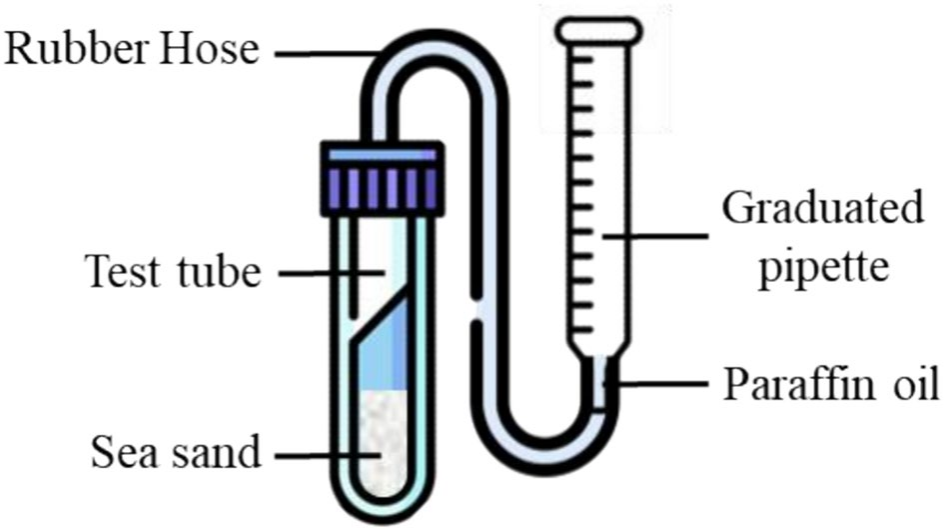
Schematic diagram of the test device.

The test setup was illustrated in Figure 3. The test steps are as follows: ① Take an appropriate amount of bacterial suspension in the logarithmic phase of growth in a centrifuge tube, centrifuge at 4000 r/min for 20 min, and after centrifugation, discard the supernatant to collect the bacterial sludge. ② Mix the bacterial pellet with an appropriate amount of denitrification medium, and use a spectrophotometer to adjust the bacterial suspension to the desired OD_600_ value. ③ Prepare sterilized sea sand with a void ratio of 0.5: based on the specific gravity of the sea sand and the target void ratio, its bulk density was calculated to be 1.77 g/cm^3^. A 25 mL graduated test tube was used in the experiment. Then, 8.85 g of sea sand was weighed into the tube and compacted to the 5 mL mark, yielding a sea-sand specimen with a void ratio of 0.5. Subsequently, 5 mL of bacterial suspension was slowly added to the tube so that the liquid level was above the sand surface. ④ The test tubes were connected to the 2 mL graduated pipettes by rubber tubing, and the ports were bonded with sealant to ensure airtightness. The bottom ports of both the rubber tubing and the graduated pipette were filled with paraffin oil. ⑤ Put the specimen into the constant temperature incubator and record the change of the liquid level on the graduated pipette every 2 h. When the liquid level stops changing, record the final scale and end the experiment. A 4-factor, 5-level test design was used in this experiment, and the test arrangement is illustrated in Table 1. The temperature was controlled by a thermostatic incubator. The initial pH of denitrification medium was about 8.2, which was adjusted by 1.0 mol HCl and NaOH solution, and was measured by pH meter (PHS-2F). OD_600_ was measured by spectrophotometer (JINGHUA-721). In order to ensure the accuracy of the test results, three sets of parallel tests were set up for each group, and all test data were taken as the average of three parallel determinations. The airtightness of the setup was verified prior to testing. Gas volume was read using a graduated pipette (resolution 0.01 mL) at consistent time intervals.

**Table 1:** Microbial gas production performance test program.

Implicit variable	Variable range	Invariant condition
Temperature (°C)	10, 15, 20, 25, 30	pH = 7, OD_600_ = 0.10, KNO_3_ = 1.0 g/L
pH	5, 6, 7, 8, 9	30 °C, OD_600_ = 0.10, KNO_3_ = 1.0 g/L
Bacterial concentration (OD_600_)	0.05, 0.10, 0.15, 0.20, 0.25	30 °C, pH = 7, KNO_3_ = 1.0 g/L
KNO_3_(g/L)	1.0, 1.5, 2.0, 2.5, 3.0	30 °C, pH = 7, OD_600_ = 0.10

The four factors (temperature, pH, OD_600_, and KNO_3_ concentration) were selected because they represent the primary controllable variables affecting denitrification-driven gas generation in MIDP systems. Temperature and pH govern microbial growth and enzyme activity, OD_600_ reflects the initial biomass level, and KNO_3_ concentration controls nitrate availability as the terminal electron acceptor. Together, these factors capture the main environmental, biological, and chemical controls relevant to coastal-sand MIDP practice.

## 3 Result and discussion

### 3.1 Effects of temperature

**Figure 4:**
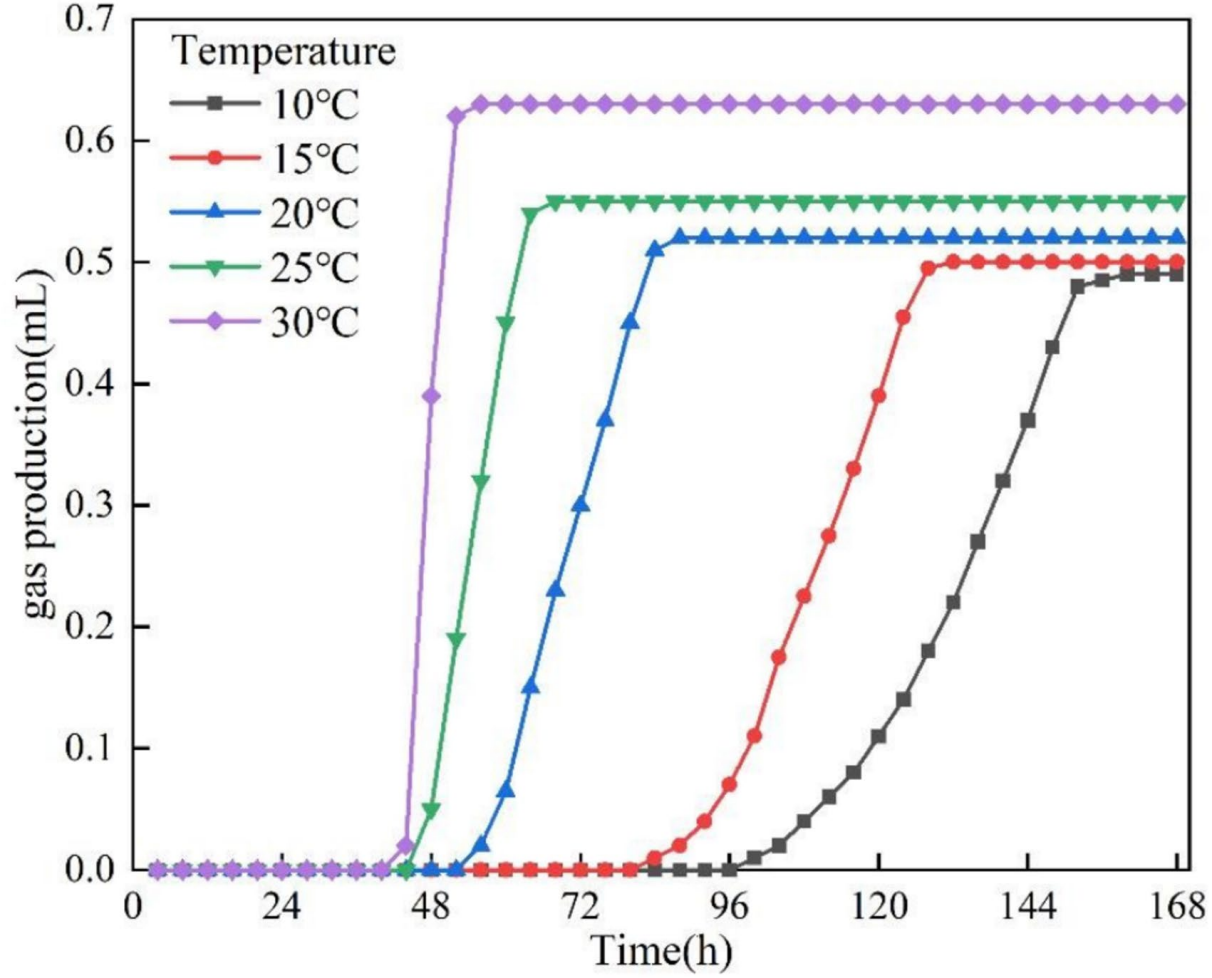
Gas production at different temperatures.

**Figure 5:**
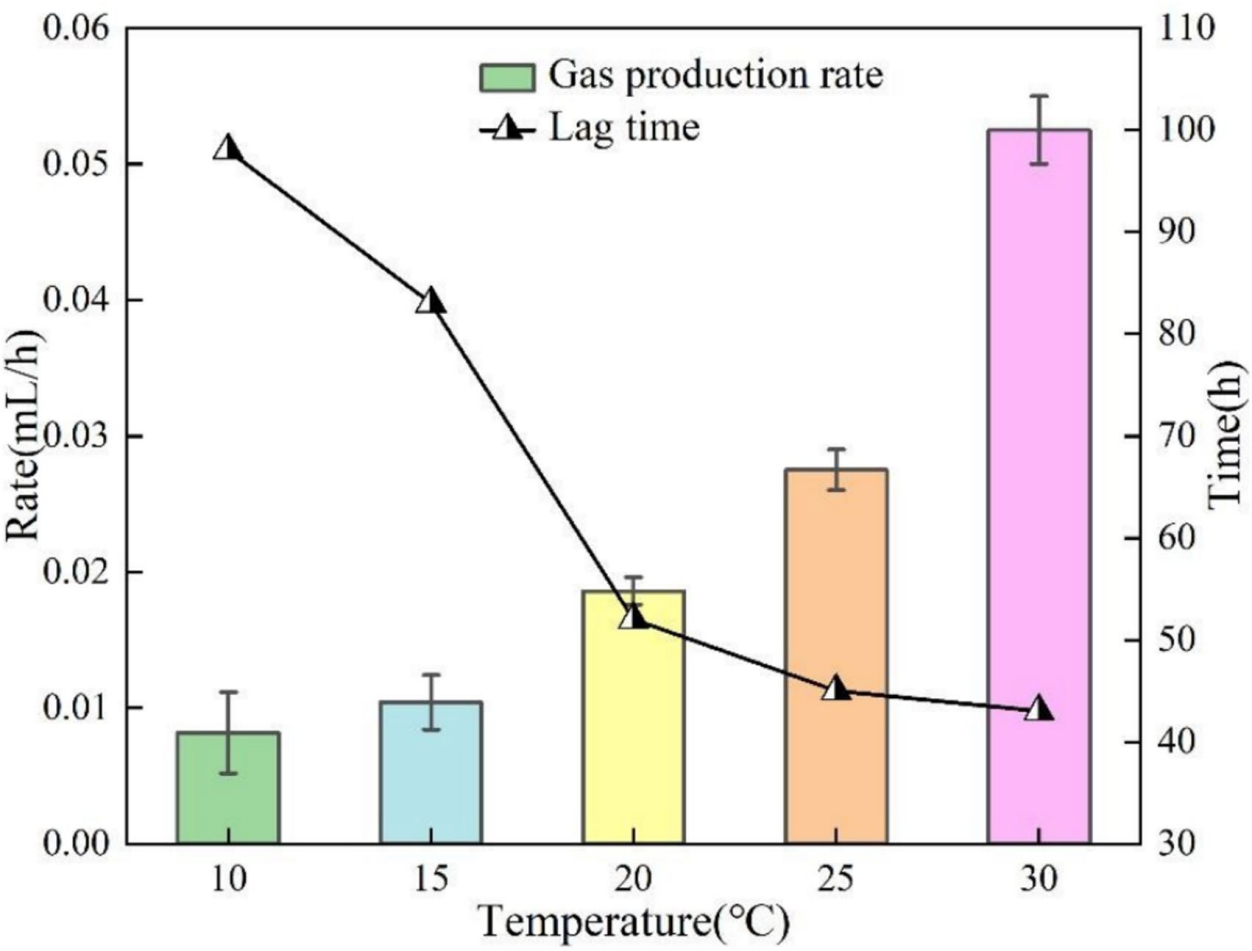
Lag time and gas production rate at different temperatures.

In this section of the test, the test conditions were fixed at pH = 9, OD_600_ = 0.1 and KNO_3_ concentration was 1 g/L except for temperature as the only variable. The gas production curves at different temperatures are shown in Figure 4. Within the temperature range of 10–30 °C, the bacteria can produce gas smoothly. With the increase of temperature, the gas production increased and the gas production time was shortened. This is due to the fact that the reduction process of NO_2_-N is strongly affected by low temperature, and the low temperature (10–15 °C) conditions have a certain inhibitory effect on the reduction process of NO_3_-N to NO_2_-N (Shan et al., 2018). The maximum gas production at 30 °C was about 0.63 mL, and the minimum gas production at 10 °C was about 0.49 mL. Lag time was defined as the time from incubation start to the onset of measurable gas accumulation in the graduated pipette. The gas production rate was calculated as the slope of the approximately linear gas-increase segment after the lag phase (Δ*V*/Δ*t*). The lag time and gas production rate at different temperatures are illustrated in Figure 5. The lag phase existed in all test groups, and the lag time was significantly shortened with increasing temperature. The longest lag time (about 98 h) was observed at 10 °C. It was shortened to 43 h at 30 °C. It is worth noting that the modulation of lag time by temperature change plateaus when the temperature rises above 20 °C. This is due to the fact that low temperatures inhibit denitrifying enzyme activity (Zhou et al., 2024). The gas production rate of the bacteria was closely related to the temperature. The test data showed that the gas production rate was 0.0082 mL/h at 10 °C, and the rate was significantly increased to 0.0525 mL/h when the temperature was increased to 30 °C, with a difference of about 6-fold. This temperature effect indicates that the microbial metabolic activity is highly sensitive to temperature conditions.

### 3.2 Effects of pH

**Figure 6:**
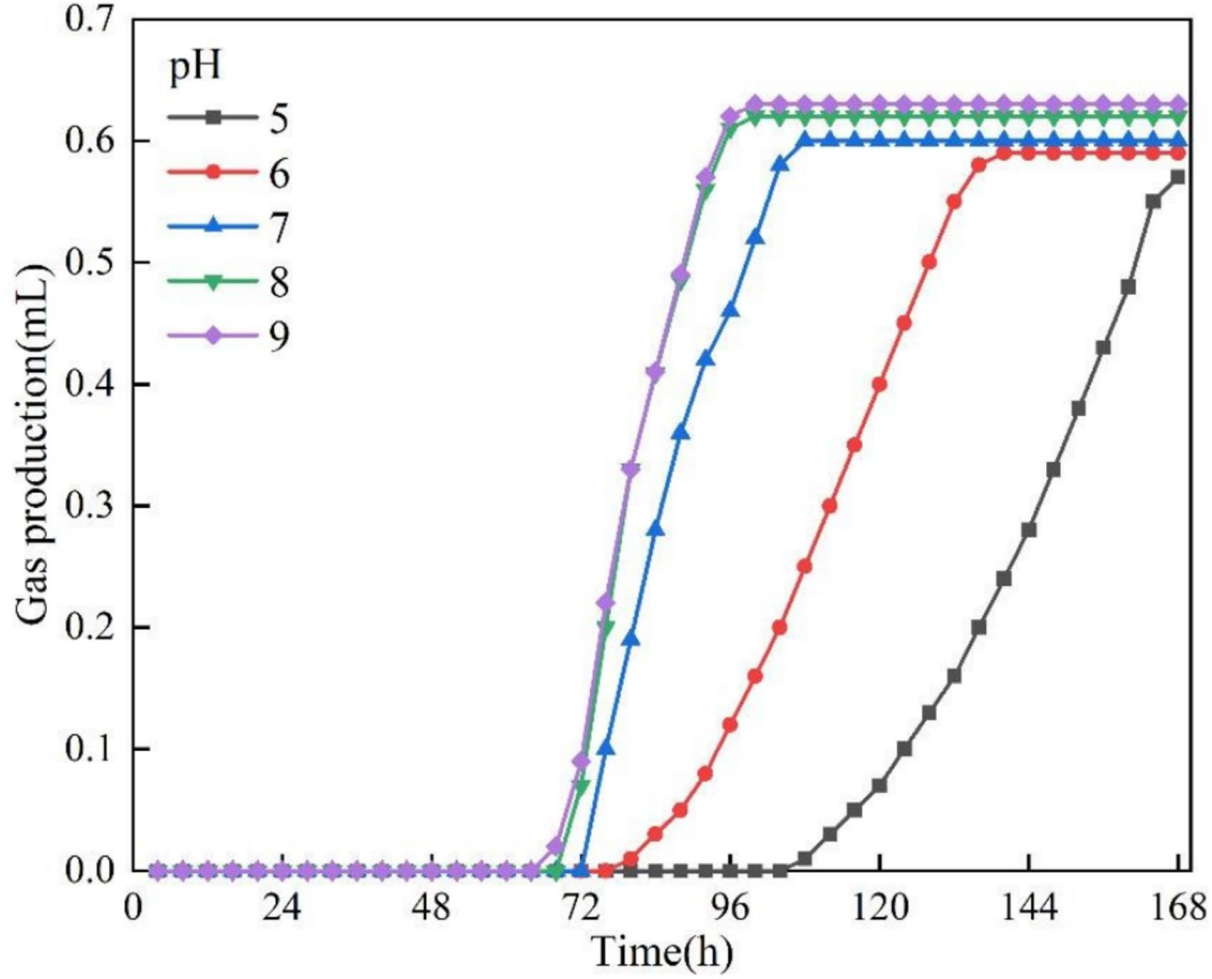
Gas production at different pH.

**Figure 7:**
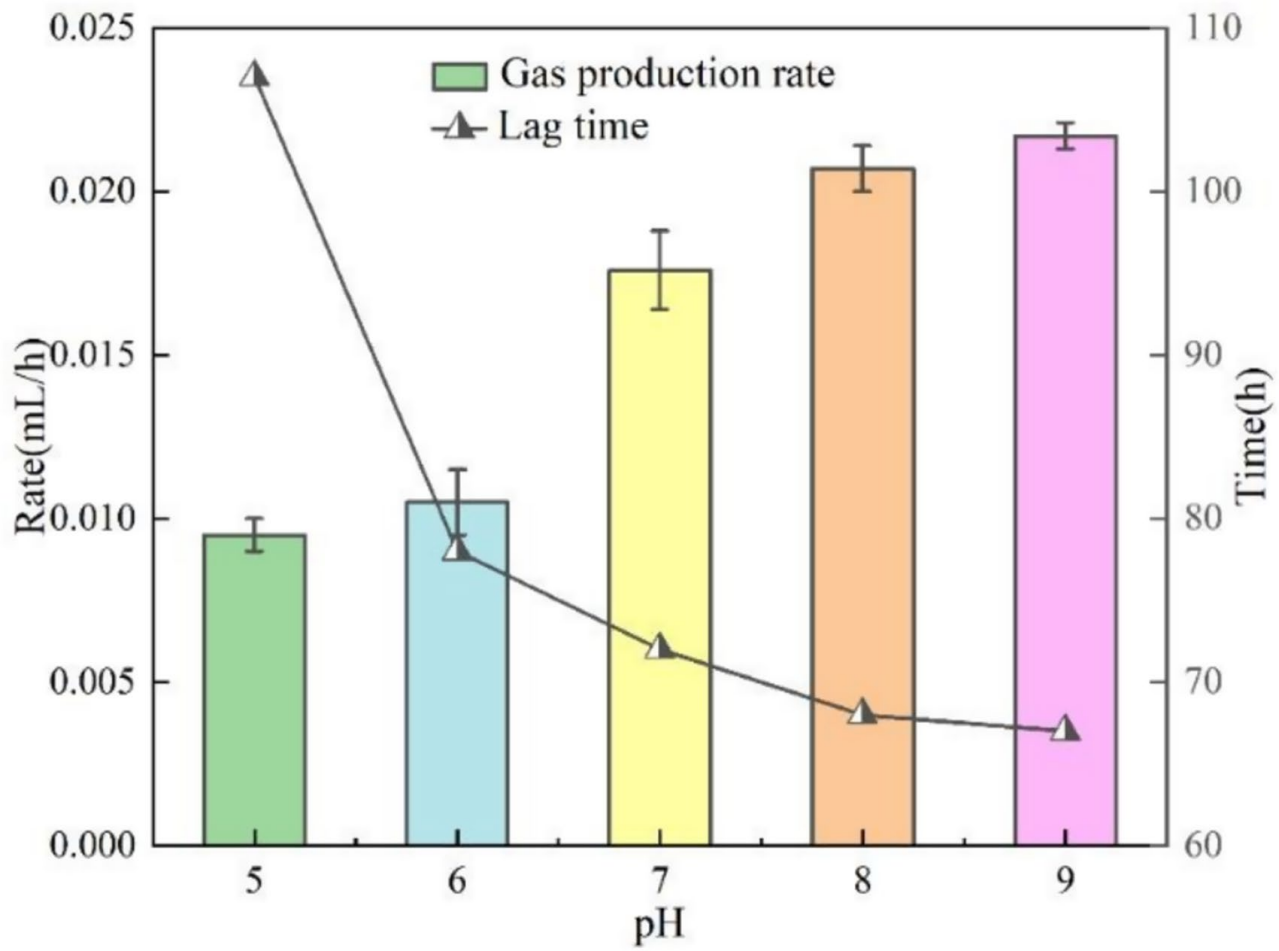
Lag time and gas production rate at different pH.

In this section of the test, the test conditions were fixed as temperature 30 °C, OD_600_ = 0.1 and KNO_3_ concentration was 1 g/L, except for pH as the only variable. The gas production curves of the bacteria at different pH are illustrated in Figure 6, and the bacteria maintained their gas production activity in the range of pH 5 to 9, and the gas production showed only a weak difference with pH fluctuation. The test data showed that the lowest gas production was about 0.57 mL at pH = 5, and peaked at about 0.63 mL at pH = 9, with a maximum difference of only 0.06 mL, indicating that the bacterium has a broader pH adaptation property. The lag time and gas production rate at different pH are illustrated in Figure 7. The lag time was negatively correlated with pH, and acidic conditions significantly prolonged the lag phase. The test data showed that the lag time reached 107 h at pH = 5, while it was shortened to 67 h at pH = 9. It is noteworthy that the modulating effect of pH change on lag time was significantly weaker in the neutral to alkaline range (pH = 7 to 9). Although pH can influence nitrite (NO₂^−^) accumulation and thus alter the early-stage kinetics (lag time and rate), the overall extent of nitrate/nitrite reduction under our test conditions remained comparable, resulting in only small differences in final gas volume (Charles and Silverstein, 1998). The bacterial gas production efficiency was significantly and positively correlated with pH. The test data showed that the gas production rate was 0.0095 mL/h at pH = 5, which was elevated to 0.0217 mL/h at pH = 9, a 2.3-fold increase. This phenomenon can be attributed to the abnormal accumulation of denitrification intermediates (nitrite) under non-suitable pH conditions (Saleema et al., 2009).

### 3.3 Effects of bacterial concentration

**Figure 8:**
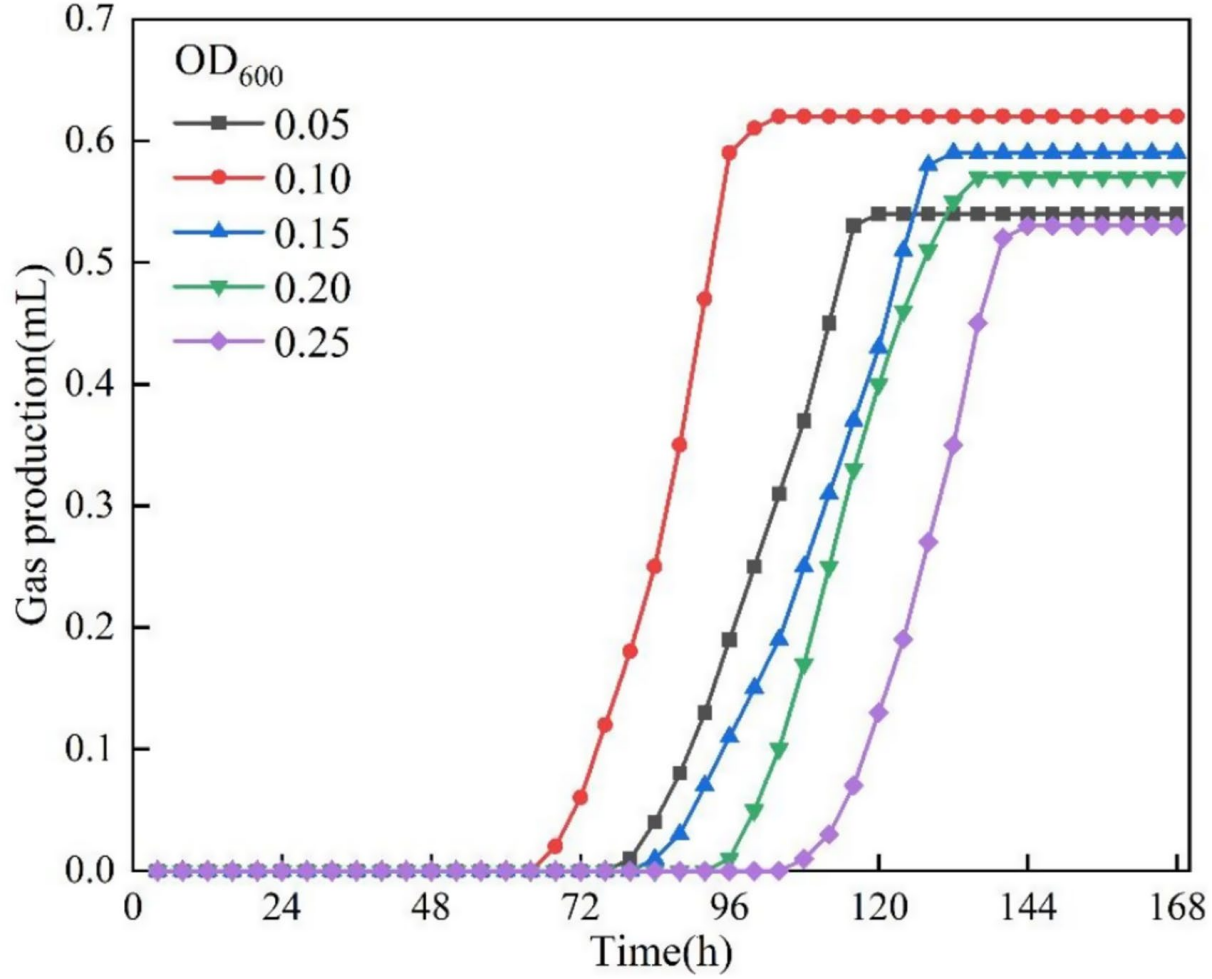
Gas production at different OD_600._

**Figure 9:**
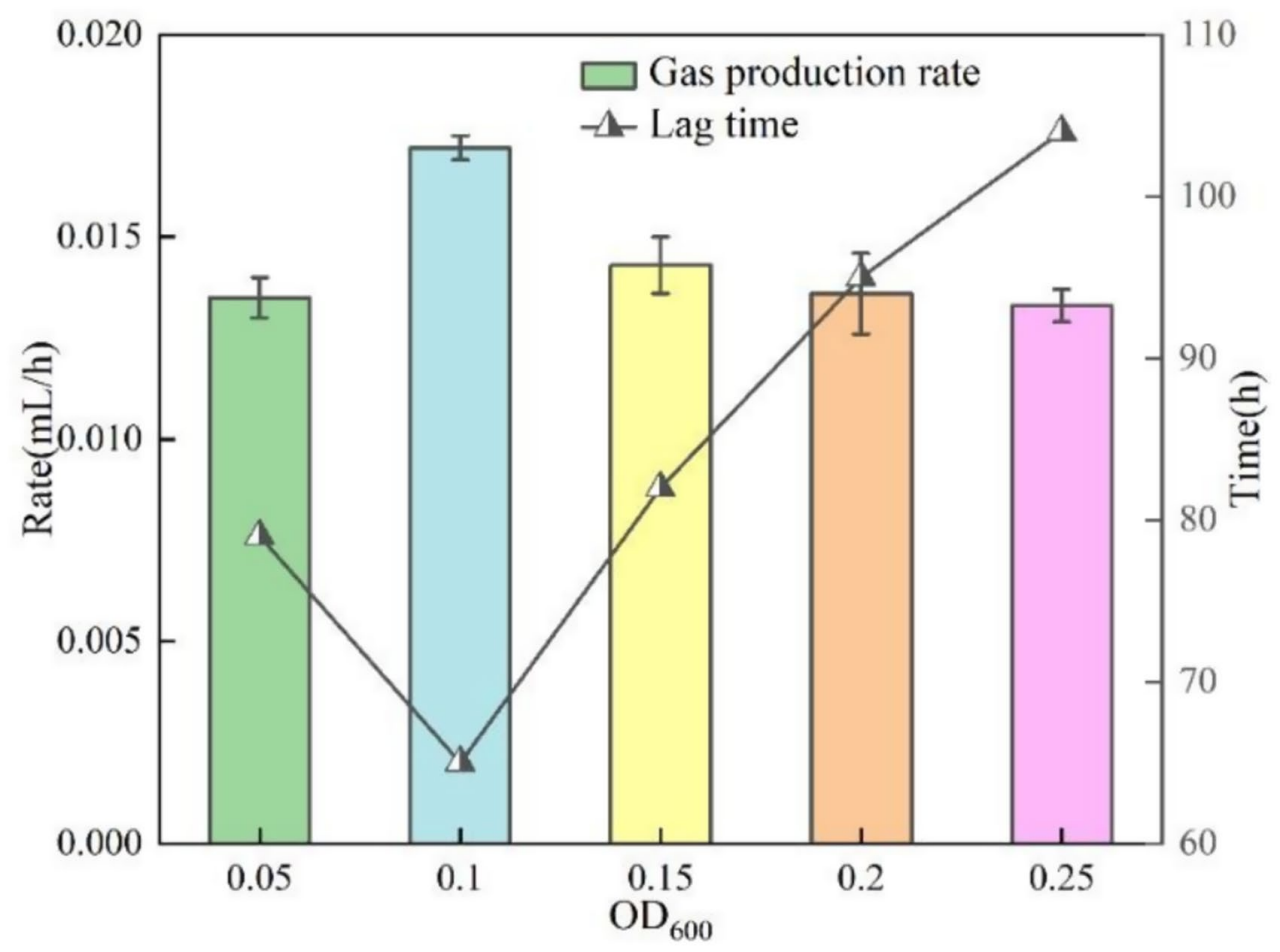
Lag time and gas production rate at different OD_600._

In this section of the test, the test conditions were fixed as temperature 30 °C, pH = 9 and KNO_3_ concentration was 1 g/L, except for the bacterial solution concentration OD_600_ as the only variable. Figure 8 demonstrates the gas production versus time curves of the bacteria at different initial concentrations. The bacterial solution optical density OD_600_ within 0.05–0.25 can maintain the gas production activity, but the gas production effect is not satisfactory when the concentration of the bacterial solution is too high or too low. The gas production peaked at 0.62 mL at OD_600_ = 0.10. The lag time and gas production rate under different OD_600_ were illustrated in Figure 9. The lag time of bacterial gas production was nonlinearly correlated with the concentration of bacterial solution, which showed a U-shape change rule of decreasing and then increasing with the increase of OD_600_. When OD_600_ = 0.10, the shortest lag time was about 65 h. With the increase of bacterial solution concentration, the rate of gas production increased firstly and then decreased, and the rate was maximum when OD600 = 0.10, which was about 0.0172 mL/h. This was due to the fact that there existed an optimal concentration value when the bacteria carried out denitrification reaction, and the concentration was lower than the value when the number of bacteria was less, and the denitrification reaction was weaker. When the concentration is higher than this value, the metabolic waste produced by the bacteria will inhibit the denitrification reaction (Ma et al., 2015), and the optimal concentration of bacteria in this test is OD_600_ = 0.10.

### 3.4 Effect of KNO_3_ concentration

**Figure 10:**
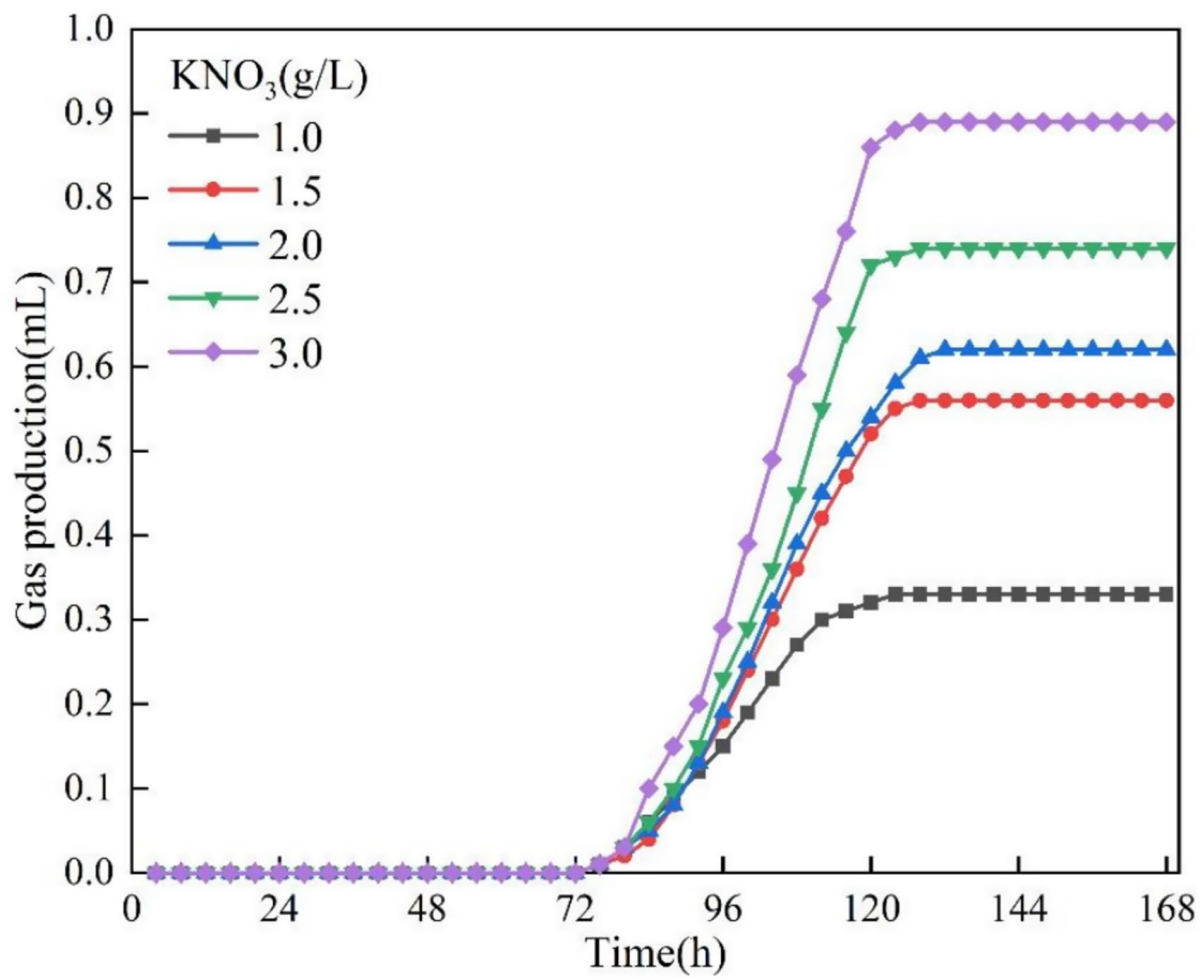
Gas production at different KNO_3._

**Figure 11:**
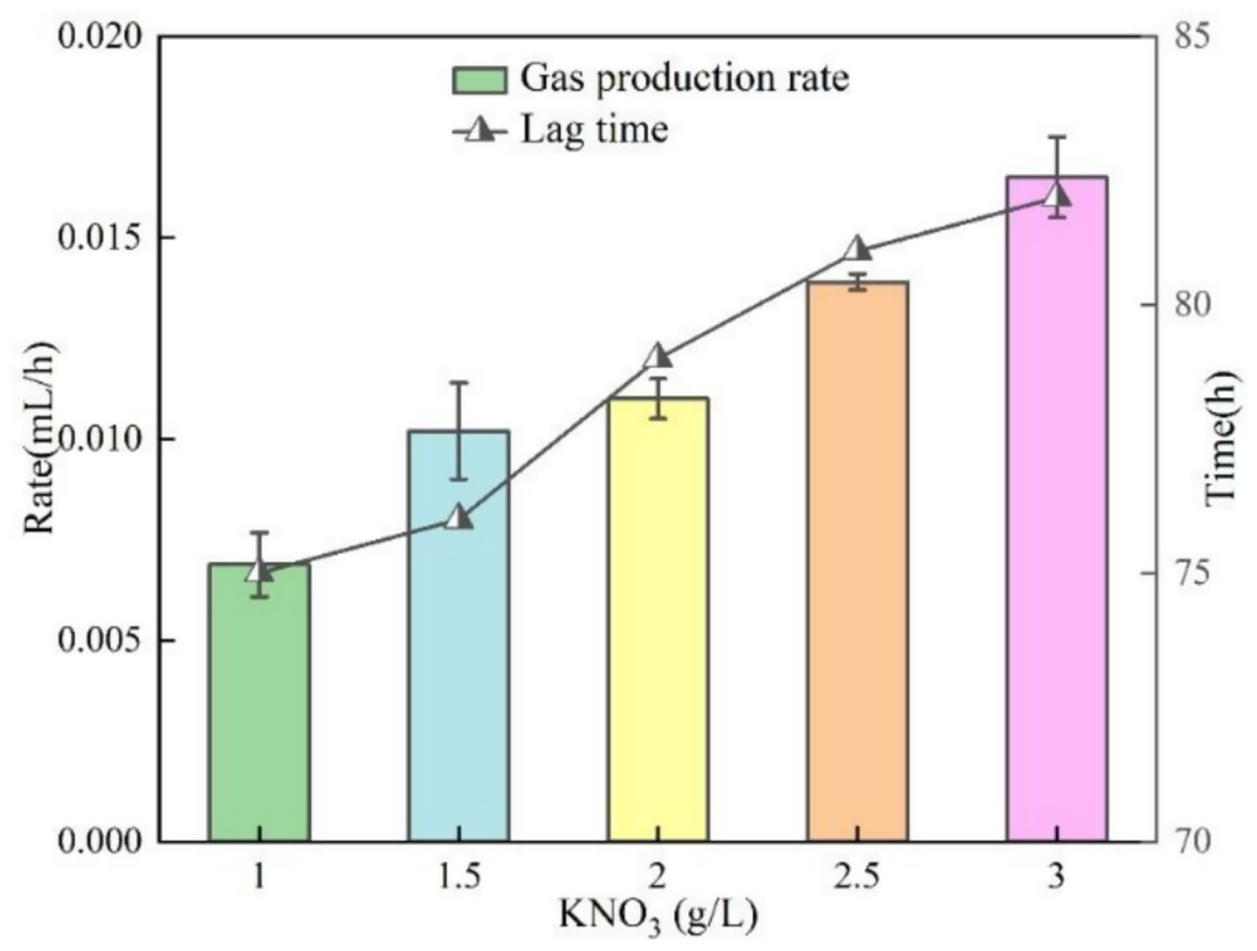
Lag time and gas production rate at different KNO_3._

In this section of the test, the test conditions were fixed at temperature 30 °C, pH = 9 and OD_600_ = 0.10, except for the KNO_3_ concentration, which was the only variable. The curves of gas production of bacteria with time at different KNO_3_ concentrations are illustrated in Figure 10. In the range of KNO_3_ concentration from 1.0 to 3.0 g/L, the bacterial gas production increased significantly with increasing concentration, while there was no significant difference in gas production time. The gas production was about 0.33 mL at a KNO_3_ concentration of 1.0 g/L and 0.89 mL at a concentration of 3.0 g/L, with an increase of about 2.5-fold in gas production, showing a significant positive concentration correlation. The bacterial stagnation time and gas production efficiency with time at different KNO_3_ concentrations is illustrated in Figure 11. The effect of KNO_3_ concentration on the reaction lag time was small, in which the longest lag time was about 72 h in the 1.0 g/L group, and the shortest was about 74 h in the 3.0 g/L group. Relevant studies have shown that DNRA and denitrification compete for nitrate, and under nitrate-limited conditions with relatively abundant electron donors, DNRA may gain a competitive advantage, thereby increasing the fraction of nitrate reduced to NH₄^+^ (Pandey et al., 2020). Therefore, the markedly reduced gas production observed at low KNO₃ in this study may be associated with a shift in nitrate-reduction pathway partitioning. The gas production rate at a KNO_3_ concentration of 1.0 g/L differed significantly from the results of the other groups of specimens, mainly because the denitrification reaction preferred the pathway of nitrite reduction to NH_4_^+^ under the low concentration condition, whereas the pathway was mainly through the generation of N_2_ at the high concentration. The difference in selectivity of the different reaction paths directly led to the significant change in gas production efficiency.

## 4 Conclusion

In this study, the gas production effect of *Pseudomonas stutzeri* in the treatment of liquefied sandy soil and its influencing factors were analyzed. Firstly, the bacterial solution with up to standard activity was obtained by culturing the bacteria, and then the key indexes such as gas production time, total gas production, gas production rate, and gas production stagnation period were tested under four conditions: temperature, pH, OD_600_, and KNO_3_ concentration. Through the analysis of test data, the influence law of different factors on the effect of gas production was clarified, and the following conclusions were finally drawn.

1. The gas production performance of *Pseudomonas stutzeri* was significantly affected by temperature in the range of 10–30 °C. With increasing temperature, the bacterial gas production increased from 0.49 mL to 0.63 mL (an increase of 39%), the lag time was shortened from 98 h to 43 h (a decrease of 56%), and the gas production rate increased from 0.0082 mL/h to 0.0525 mL/h (a 6.4-fold enhancement). Temperature affects the metabolic process mainly by regulating the activity of denitrifying enzymes, and the efficiency of denitrification reaction tends to stabilize when the temperature exceeds 20 °C.
2. In the range of pH = 5 to 9, pH had a small effect on the gas production of *Pseudomonas stutzeri*, 0.63 mL at pH = 9 and 0.57 mL at pH = 5, an increase of 10.5%. Acidic conditions (pH = 5) prolonged the lag phase to 107 h, a 37% increase over alkaline conditions (pH = 9). Meanwhile, the alkaline environment significantly enhanced the gas production rate to 0.0217 mL/h, which was 2.3 times higher than that of the acidic condition (0.0095 mL/h). pH affects the reaction process by regulating the rate of accumulation of nitrite (NO^2−^) intermediates, and an acidic environment leads to prolonged NO^2−^retention time, thus delaying denitrification. However, complete denitrification could be accomplished in different pH systems.
3. When OD_600_ is between 0.05 and 0.25, the gas production performance shows a trend of increasing and then decreasing. The optimal gas production was achieved at OD_600_ = 0.10, where the gas production volume (0.62 mL) and the gas production rate (0.0172 mL/h) were maximized, while the stagnation period was shortened to 65 h. When the concentration of bacteria was too low (OD_600_ < 0.10), the intensity of reaction was limited due to insufficient biomass. When the concentration was too high (OD_600_ > 0.10), there was an inhibitory effect due to the accumulation of metabolic by-products, suggesting that there is a clear population density regulation threshold for the bacterial denitrification process.
4. Increasing KNO_3_ concentration (1.0–3.0 g/L) significantly increased gas production (2.5-fold from 0.33 mL to 0.89 mL), but had limited effect on lag and gas production time. Nitrite intermediates were preferentially reduced via the NH_4_^+^ pathway (63.2% of the total product) rather than the gaseous N_2_ generation pathway, and this difference in metabolic flux allocation resulted in a lower gas production efficiency.

## Data Availability

The original contributions presented in the study are included in the article/supplementary material, further inquiries can be directed to the corresponding author.
